# Synthetic mechanoreceptor engineering: From genetic encoding to DNA nanotechnology-based reprogramming

**DOI:** 10.1016/j.mbm.2025.100160

**Published:** 2025-10-06

**Authors:** Sihui Yang, Zhou Nie

**Affiliations:** a School of Pharmaceutical Science, Hengyang Medical School, University of South China, Hengyang, 421001, Hunan, China; b State Key Laboratory of Chemo/Biosensing and Chemometrics, Hunan Provincial Key Laboratory of Biomacromolecular Chemical Biology, College of Chemistry and Chemical Engineering, College of Biology, Hunan University, Changsha, China

**Keywords:** Synthetic mechanoreceptors, DNA nanotechnology, Non-genetic engineering, Mechanotransduction regulation

## Abstract

Precise modulation of mechanoreceptor-mediated signal transduction is crucial for decoding cellular mechanotransduction mechanisms and programming cell fate. This review provides a comprehensive summary of recent advances in engineering synthetic mechanoreceptors, spanning from protein-centric genetic encoding to DNA nanotechnology-based non-genetic reprogramming strategies. Genetic engineering strategies employ protein structure encoding and site-directed mutagenesis to reprogram force-response functions in natural mechanoreceptors. As a complementary non-genetic approach, DNA nanotechnology leverages its programmability, modularity, and predictable mechanical properties to achieve precise control over receptor functionalities. The flourishing development of DNA mechanosensitive nanodevices has provided a promising synthetic toolkit for manipulating mechanoreceptors, enabling precise control over receptor spatial organization and signal transduction. A key innovation is the development of novel DNA-functionalized artificial mechanoreceptors (AMRs), which confer force-responsiveness to naturally non-mechanosensitive receptors without genetic modification, thereby enabling customized mechanotransduction and mechanobiological applications. Collectively, this paradigm shift highlights DNA-based non-genetic receptor engineering as a versatile and powerful toolkit, paving new avenues for mechanobiology research and pioneering force-directed therapeutic strategies in regenerative medicine.

## Graphical abstract

**Figure:**
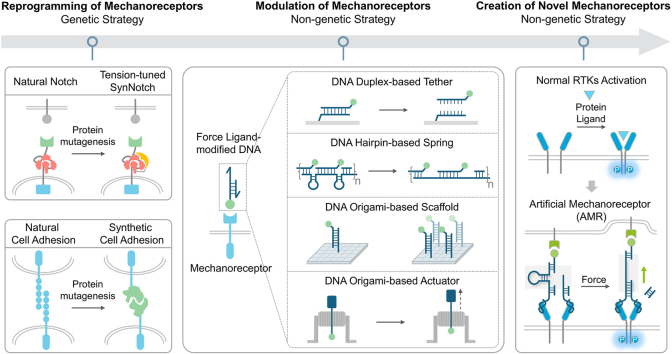

## 1 Introduction

Mechanical forces play pivotal roles in fundamental biological processes, from growth and development to the progression of various pathological conditions, including tissue fibrosis, cardiovascular diseases, and cancer.^1^, ^2^, ^3^, ^4^, ^5^, ^6^ As the basic structural and functional units of organisms, cells are continuously exposed to mechanical stimuli generated by their surrounding extracellular matrix, neighboring cells, and fluidic tissue environments.^7^, ^8^, ^9^, ^10^ These physical signals are often detected by specialized mechanoreceptors on the cell surface and transduced into biochemical signals, thereby regulating intracellular signaling pathways and ultimately determining cellular fate and behaviors, including cell adhesion, migration, proliferation, differentiation, development, and immune responses.^11^, ^12^, ^13^ Reprogramming cellular mechanosensing and mechanotransduction through the engineering of receptors offers a powerful strategy to gain deeper insights into mechanobiology and to develop novel force-directed cell therapies, holding significant promises for advancing neurobiology, immunology, and regenerative medicine.

**Fig. 1:**
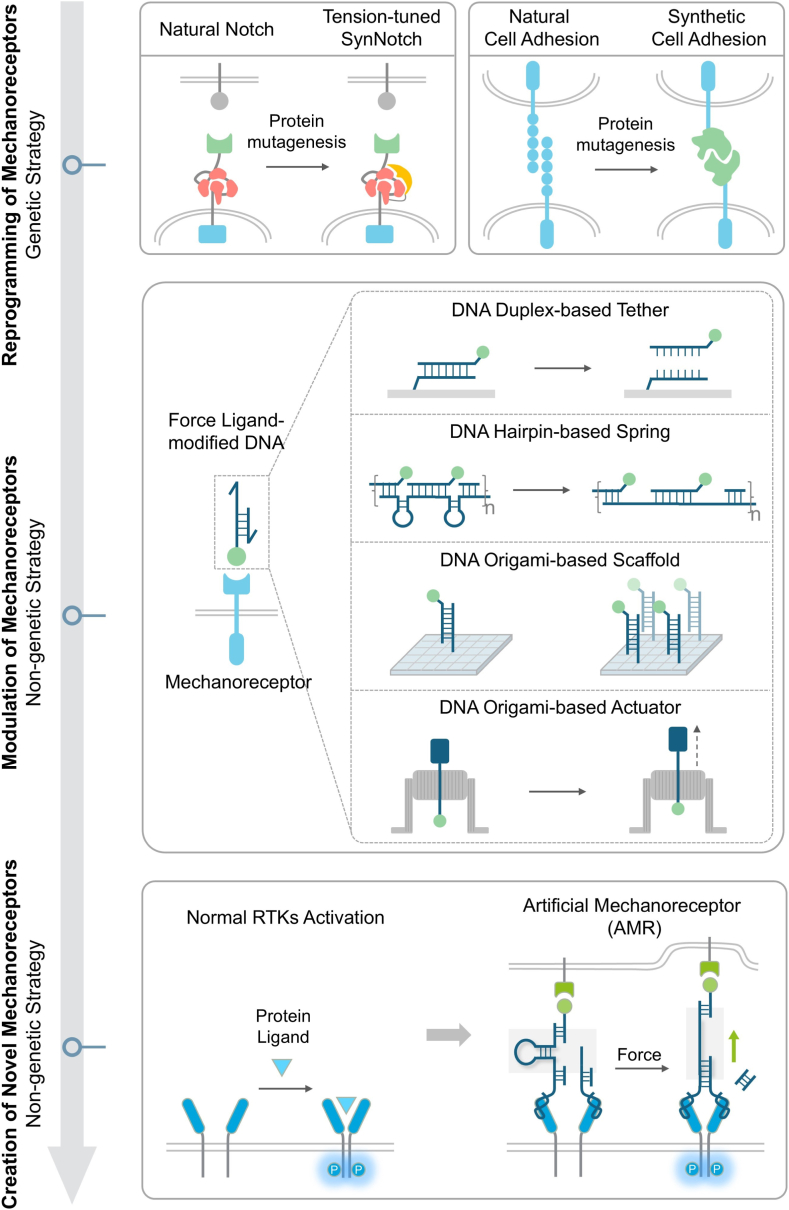
Recent advances from genetic to non-genetic strategies in receptor-based mechanotransduction engineering. Top: The genetic approaches rely on the reprogramming of natural mechanoreceptors by protein structure fusion and directed mutagenesis to achieve tunable mechanical sensitivity (SynNotch) and novel molecular recognition (SynCAMs). Middle: The non-genetic approaches leverage DNA mechanosensitive nanodevices for receptor modulation without altering genetic sequences. Approaches include DNA duplex-based tether, DNA hairpin-based spring, DNA origami-based scaffold and actuator for spatial organization and controlled activation of natural mechanoreceptors. Bottom: A de novo non-genetic approach enables the construction of DNA-functionalized artificial mechanoreceptors (AMRs) for reprogramming non-mechanoresponsive receptors (RTKs) to confer force responsiveness.

During mechanotransduction, natural mechanoreceptors (including integrins, cadherins, Notch, T cell receptors, etc.) undergo force-induced conformational changes, such as protein unfolding or deformation.^14^ During this process, mechanical signals are converted into biochemical signals, which propagate further downstream within the cells. User-defined engineering of mechanoreceptors enables precise reprogramming of mechanical signaling, thereby allowing directed manipulation of cellular behaviors. To date, significant progress has been made in the synthetic engineering of natural mechanoreceptors. For instance, synthetic Notch receptors (synNotch) and synthetic cell adhesion molecules (synCAMs) utilize genetic engineering of natural Notch receptors and adhesion receptors to achieve force-induced specific gene expression and redirection of native adhesion signals, respectively (Fig. 1).^15^^,^^16^ These synthetic biology-driven approaches enable customized recognition of novel mechanical inputs and cellular biochemical reactions by genetically reconstructing mechanoreceptors, thereby effectively modulating cell signaling pathways. However, despite these successes, generalized engineering approaches for mechanoreceptors remain constrained by the limited variety of natural mechano-switch scaffolds and the inability to reprogram other non-mechanoresponsive receptors to confer customized mechanosensing capabilities. This limitation likely stems from the absence of generalizable native mechanotransduction mechanisms, as natural mechanoreceptors operate through highly diverse and specialized principles that are not yet fully elucidated. Furthermore, fine-tuning mechanical sensitivity remains challenging due to the requirement for iterative trial-and-error optimization of complex protein architectures. Critically, reliance on genetic approaches may introduce additional problems caused by inherent potential complications and challenges associated with exogenous gene transfection and expression.^17^^,^^18^ Therefore, there is a pressing need to develop non-genetic receptor engineering strategies independent of natural mechanosensing mechanisms, which offer user-programmable mechanical signal manipulation capabilities to precisely reprogram cell functions.

DNA-based nanodevices, leveraging excellent stimuli-sensitivity, programmability, and predictability, are emerging as powerful tools for engineering receptors and manipulating mechanical signaling pathways in a non-genetic way.^19^, ^20^, ^21^, ^22^, ^23^, ^24^ DNA mechanosensitive nanodevices, e.g., DNA duplex-based tether, DNA hairpin-based spring, DNA origami-based scaffold and actuator, have provided diverse nanoscale mechanosensing and mechano-modulation modules, enabling engineering of recognition specificity and precision manipulation for mechanoreceptors (Fig. 1).^25^, ^26^, ^27^, ^28^ They facilitate dynamic control over mechanical ligand presentation, mechanoreceptor spatial organization, and cellular signaling pathway activation, thereby accomplishing regulation of cellular mechanosensing, signal transduction, and behavioral responses. Building upon this foundation, a recent work introduced a novel concept of artificial mechanoreceptor (AMR), which aims to utilize generalized mechanosensing-and-transmitting DNA (GMD) nanodevices as plug-and-play molecular tools to functionalize non-mechanoresponsive receptor tyrosine kinase (RTKs),^29^, ^30^, ^31^ thereby enabling force-responsive control of cells (Fig. 1).^32^ This study demonstrated the non-genetic strategy capable of converting non-native mechanosensitive pathways into force-responsive signal pathways, enabling customized mechanotransduction reprogramming and tailored cellular mechanical responses, which represents a distinctive advancement in mechanobiology.

In this review, we discuss the latest advancements in cellular mechanotransduction reprogramming, delineating a paradigm shift from protein-centric genetic approaches to DNA nanotechnology-enabled non-genetic strategies. Natural mechanoreceptors exhibit force-dependent signaling characteristics, while genetically programmed synthetic receptors employ protein structure fusion and directed mutagenesis to precisely reprogram receptor responses to user-defined mechanical cues, opening new avenues for mechano-based synthetic biology and immunotherapy. However, genetically engineered mechanoreceptors remain constrained by inherent limitations, including high trial-and-error costs, incompatibility with non-mechanosensitive receptors, and the absence of generalizable mechanotransduction principles. In contrast, the intrinsic advantages of DNA nanotechnology endow it with unique applications in cellular mechanoreceptor regulation in a non-genetic way. Crucially, DNA-based mechanosensitive nanodevices enable dynamic remodeling of cellular mechanotransduction without genomic alteration, achieving precise control over receptor clustering, ligand presentation, and downstream pathway activation. A prime example is the development of de novo DNA-functionalized AMRs independent of natural mechanosensory mechanisms, which confer RTKs with force sensing and transduction functions. We posit that these DNA-based mechanoreceptor reprogramming strategies will pioneer novel pathways for mechanobiology research and biomedical applications.

## 2 Natural mechanoreceptors

**Fig. 2:**
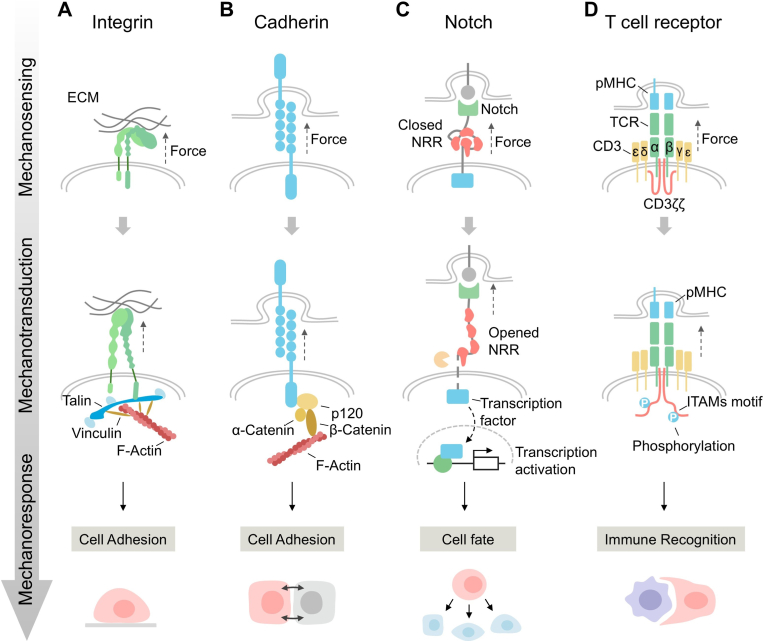
Mechanotransduction mechanism of natural mechanoreceptors. (A) Integrin: Mechanical forces acting on the extracellular domain of integrins induce their transition from a low-affinity to a high-affinity state. The activated integrins then couple with the actin cytoskeleton through adaptor proteins (talin and vinculin) to form focal adhesions, which further initiate downstream signaling pathways. (B) Cadherin: E-cadherin mediates calcium-dependent homophilic binding via its extracellular domain with E-cadherin on adjacent cells. Its intracellular domain binds to catenin family proteins (catenin and p120), thereby linking to the actin cytoskeleton. Mechanical forces are transmitted through the cytoskeleton to regulate adhesion strength and signaling. (C) Notch: Ligands (Delta) bind to the extracellular domain of Notch and exert pulling forces, leading to conformational changes in the NRR that expose protease cleavage sites. This results in the release of the intracellular domain into the nucleus, where it acts as a transcription factor to activate target gene expression. (D) Upon TCR binding to pMHC, mechanical pulling forces are applied. These forces induce conformational changes in the TCR, exposing ITAMs (immunoreceptor tyrosine-based activation motifs). The ITAMs are then phosphorylated, initiating downstream signaling pathways.

Among all forms of life, the foundation of survival lies in the ability to adapt to environmental mechanical forces. Force plays a crucial role in the formation, development, and maintenance of tissues and organs.^33^ Nearly all organisms have evolved force-sensitive structures spanning macroscopic (e.g., organs, tissues), microscopic (e.g., cells), and nanoscopic (e.g., molecules, proteins) scales to perceive mechanical forces, including compression, tension, shear stress, and hydrostatic pressure.^34^ Cells within tissues typically generate substantial mechanical forces at the nanonewton (nN) level.^35^ In contrast, individual mechanoreceptors on the cell surface experience much smaller forces, typically with piconewton (pN) resolution.^36^, ^37^, ^38^, ^39^ These molecular-level forces enable precise spatiotemporal regulation of signal transduction processes, thereby directly or indirectly controlling a series of biological responses such as cell differentiation, gene expression, and apoptosis. When sensing force inputs from the matrix or neighboring cells, natural mechanoreceptors undergo force-induced conformational changes and trigger mechanical transduction processes to regulate fundamental cellular functions and responses. This article mainly focuses on mechanoreceptors, including cell adhesion receptors, such as integrins and cadherins, Notch receptors, and T cell receptors (Fig. 2).

### 2.1 Integrins

Integrin-mediated mechanotransduction plays a pivotal role in growth, development, and tissue homeostasis.^40^, ^41^, ^42^ Integrins are heterodimeric transmembrane complexes composed of α and β subunits, with each αβ pair exhibiting specific binding specificity and signaling characteristics.^43^ They transmit mechanical signals from the extracellular environment into the cell by binding to extracellular matrix (ECM) proteins such as collagen, fibronectin, and laminin. Additionally, certain integrins are responsible for binding to cell surface proteins. For example, the integrin family member lymphocyte function-associated antigen-1 (LFA-1) can specifically bind to intercellular adhesion molecule-1 (ICAM-1) on adjacent cells, promoting cell adhesion in immune and inflammatory responses.^44^, ^45^, ^46^ During mechanotransduction, mechanical forces induce conformational changes in the extracellular domain of integrins, causing them to transition from a low-affinity state to a high-affinity state (Fig. 2A). This activation enables coupling to the actin cytoskeleton via adaptor proteins and focal adhesion proteins, initiating downstream signaling pathways, thereby regulating cellular long-term changes and gene expression.^47^

### 2.2 Cadherins

Cadherins are a class of calcium-dependent transmembrane proteins that primarily mediate cell-cell adhesion, which play a crucial role in various morphogenetic processes, including cell sorting, rearrangement, and migration.^48^ E-cadherin, as a type I cadherin, is the most extensively studied cadherin, widely expressed in epithelial cells, and possesses both adhesion and signal transduction functions. During cell adhesion, E-cadherin engages in calcium-dependent homophilic interactions with E-cadherin molecules on neighboring cells and forms cadherin-catenin complexes within the cell (Fig. 2B).^49^ In these adhesion complexes, the intracellular domains of E-cadherins recruit adapter proteins like p120 and catenin, which engage the cortical actin cytoskeleton and therefore drive cellular expansion.^50^ This molecular architecture not only stabilizes intercellular junctions but also integrates mechanical and biochemical signals to regulate cell behaviors.

### 2.3 Notch receptors

Notch receptors are single-channel transmembrane proteins that play a crucial role in determining the fate and function of diverse cell types.^51^ They contain a recognition domain for binding neighboring cell membrane ligand Delta, and a negative regulatory region (NRR) which undergoes force-induced conformational changes upon ligand engagement.^52^, ^53^, ^54^ Specifically, the application of physiologically relevant mechanical force disrupts the NRR structure, exposing a protease cleavage site and leading to the release of intracellular domains (Fig. 2C). The intracellular domains then function as transcription factors and, upon release from the membrane, directly enter the cell nucleus to regulate target gene expression. Therefore, by translating external receptor-ligand mechanical interactions into internal transcriptional responses, the Notch signaling pathway plays pivotal regulatory roles in development, immunity, tissue homeostasis, and other complex physiological processes.^55^^,^^56^

### 2.4 T cell receptors

T cell receptors (TCRs), predominantly composed of α and β chains, typically form TCR complexes with six associated non-polymorphic chains that play pivotal roles in T cell function and immune synapse formation.^57^^,^^58^ T cell activation exhibits good specificity, as signal initiation requires TCR recognition of antigen peptides presented by major histocompatibility complexes (pMHC) on antigen-presenting cells (APCs).^59^ Mechanical force is an indispensable component of TCR-APC interactions and a key determinant of pMHC-triggered TCR activation.^60^^,^^61^ Forces at the TCR-pMHC binding interface facilitate the formation of stable catch bonds between TCR and pMHC complexes, which propagate along the receptor into the cell, inducing conformational changes that expose immunoreceptor tyrosine-based activation motifs (ITAMs) for phosphorylation, thereby initiating biochemical signaling (Fig. 2D).^62^, ^63^, ^64^ Elucidating the mechanical sensing mechanisms of TCRs enables incorporation of biomechanical principles into next-generation T cell-based immunotherapies. This knowledge informs rational design strategies for engineering TCRs and chimeric antigen receptors (CARs), advancing biologically optimized approaches to modulate T cell immunity.

### 2.5 Mechanosensing mechanisms of mechanoreceptors

Mechanical stimuli typically activate cell mechanotransduction through interactions between force-related ligands and specific force-sensitive receptors on the cell membrane. Upon ligand engagement, mechanoreceptors transmit mechanical signals across the cell membrane to initiate downstream mechanical responses. This process involves three sequential steps:^14^^,^^34^

Step 1 Mechanosensing: Force-related ligands bind to mechanoreceptors on the cell surface and apply physical forces.
Step 2 Mechanotransduction: Mechanical signals are converted into biochemical signals. This often involves force-induced conformational changes in specific receptor domains, leading to exposure of hidden signal molecule binding sites or enzymatic cleavage sites, thereby initiating intracellular signaling cascades.
Step 3 Mechanoresponse: Changes in cellular function, including short-term integrated signal responses of the locomotor system (e.g., cytoskeletal reorganization) and long-term alterations in cellular molecular composition (e.g., gene expression reprogramming).

## 3 Genetically engineered mechanoreceptors

**Fig. 3:**
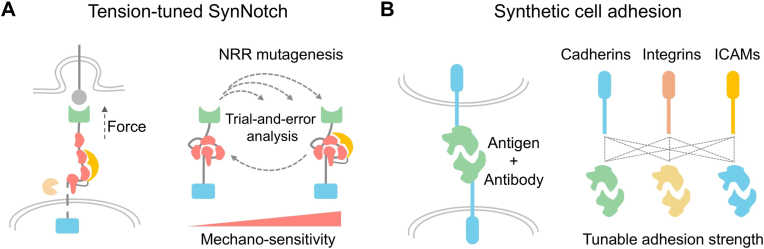
The conceptual design of SynNotch and synCAMs from natural mechanoreceptors via genetic approaches. (A) SynNotch enables rational design and modification of the interface between the NRR and the NRR-binding antibody fragment via site-directed mutagenesis, enabling precise tuning of its force-responsive threshold. (B) SynCAMs replace the extracellular domains of natural adhesion receptors (such as cadherins, integrins, and ICAMs) with heterologous binding domains exhibiting “orthogonal” interactions, thereby achieving user-defined cell recognition specificity and reprogramming the spatial organization of cells.

A key research focus in mechanobiology is the redesign of mechanoreceptor-mediated signaling pathways to precisely control cell fate or program immune responses. Genetic engineering is an intuitive and practical technical approach that enables precise control of cell surface receptors for cellular behavior regulation.^65^^,^^66^ Genetically engineered synthetic receptors have been widely applied to achieve synthetic control over customized receptor activation and downstream signal transduction, rewiring endogenous signaling pathways in response to new inputs.^67^^,^^68^ Currently, there are only two examples of genetically engineered synthetic mechanoreceptors, e.g., synNotch and synCAMs (Fig. 3).^15^^,^^16^ They are genetically engineered from their natural cognate mechanoreceptors, Notch and cadherin, respectively. These synthetic mechanoreceptors both respond to cell-cell interactions by sensing customized ligand-mediated traction/tension force input, subsequently regulating specific downstream signaling pathways for regulating immunotherapy and tissue patterning.

### 3.1 Synthetic Notch receptors (synNotch)

In nature, the Notch signaling pathway plays a crucial role in translating external receptor-ligand interactions into internal transcriptional responses. Upon binding to its ligand, Notch undergoes a conformational change, triggering an enzymatic cleavage reaction that releases the intracellular domain, which then translocates to the nucleus to regulate gene expression. By swapping the extracellular and intracellular modules of Notch with diverse recognition domains (antibody or peptide-based) and transcriptional activators, respectively, synNotch can be customized to recognize different extracellular ligands, leading to the activation of specific intracellular genes.^69^ A significant advancement is the development of the tension-tuned synNotch receptor with tunable mechanical sensitivity via integration of the NRR with an NRR-binding antibody fragment and modulating the binding interface of NRR/NRR-binding antibody by structure-guided mutagenesis (Fig. 3A).^15^ Through strategic protein structure mutagenesis, a suite of receptors with force-response thresholds spanning the physiological pN range was developed. Cells expressing these engineered receptors can discriminate between different tension magnitudes and respond through predefined transcriptional programs. This work provided another regulatory layer on tensile force sensitivity to traditional synNotch, which empowers cells to detect a variety of tensile stresses, enabling customizable transcriptional programs in response to specific ligand and tensile force levels.

### 3.2 Synthetic cell-adhesion molecules (synCAMs)

Cell adhesion, another crucial mechanism in mechanical interactions between cells, has become a focal point in synthetic biology research. Specifically, by engineering cell adhesion receptors, researchers can precisely control cellular recognition, spatial organization, intercellular connections, and even tissue morphogenesis. Lim et al. developed a series of synCAMs by combining the intracellular domains of native adhesion molecules (such as cadherins, integrins, and ICAMs) with extracellular domains featuring orthogonal interactions (Fig. 3B).^16^ These synCAMs enable customizable cell-cell interactions while maintaining adhesion properties comparable to natural molecules. The characteristics of the intracellular domains determine the morphology and mechanical properties of cell interfaces, whereas distinct extracellular interaction domains independently dictate the connection patterns between cells. This orthogonal adhesion molecular toolbox not only makes programmable assembly of multicellular structures possible but also provides novel approaches for systematic reconstruction of native tissues. The ability to systematically regulate intercellular adhesion offers powerful tools for developmental biology, neuroscience, and immunology research, while simultaneously creating new application prospects for multicellular tissue repair and therapeutic cell design.

### 3.3 Challenges in synthetic mechanoreceptors

Synthetic mechanoreceptors enable cells to detect, process, and respond to artificially defined mechanical signals. These technologies facilitate customized sensing and response programs, linking cellular interactions with extracellular mechanical cues to user-defined reactions. The development of synNotch/synCAMs relied on the mechanotransduction mechanisms of natural Notch/CAMs and swapping extracellular input/intracellular output with diverse customized recognition/effector domains. Because of the structural sophistication of natural mechanoreceptors and their complicated and still elusive mechano-responsive mechanisms, currently available genetically engineered synthetic mechanoreceptors all depend on the modifications of natural mechanoreceptors. Both de novo design of the mechanotransduction process and reprogramming a non-mechanoreceptor with force-responsive function are highly challenging and still not explored to date, probably due to the force-responsive mechanism of a mechano-receptor being highly specific and hardly compatible and expandable to other receptor-based signaling mechanisms. Moreover, due to the structural complexity of protein-based mechanoreceptors, chimeric construction via genetic engineering requires a meticulous analysis of the receptor structure, a profound preliminary study on the receptor response mechanism, and a substantial amount of trial-and-error in mutations to regulate the function of mechanoreceptors. Additionally, genetic receptor engineering faces inherent limitations, including interference and unpredictability caused by exogenous gene introduction, as well as potential adverse effects and safety risks associated with viral vector systems. These limitations underscore the need for alternative, adaptable, and non-genetic mechanoreceptor reprogramming strategies capable of rewiring receptor specificity in response to mechanical cues, thereby regulating biochemical signal transduction and cellular behaviors without requiring genetic engineering.

## 4 DNA-based molecular tools for mechanical sensing and regulation

Non-genetic engineering, especially those based on DNA nanotechnology, serves as a ready-to-use option suitable for the on-demand functionalization of various cells expressing the receptors of interest without necessitating tailored genetic modifications. DNA has been widely employed in the development of functional nanodevices due to its programmable nature and highly predictable self-assembly capabilities.^70^, ^71^, ^72^, ^73^ In recent years, various DNA-based mechanosensitive nanodevices have been engineered to investigate mechanical processes at the molecular scale.^26^^,^^28^^,^^74^, ^75^, ^76^, ^77^ These nanodevices have significantly advanced our understanding of cellular mechano-response mechanisms. Notably, DNA mechanosensitive nanodevices exhibit unique advantages, including simple design principles, high versatility, precise tunability, favorable mechanical rigidity, and programmable mechanical properties.^27^ These features make them particularly well-suited for modulating cellular mechanical functions via membrane receptor manipulation.

### 4.1 DNA-based mechanical probes

Receptor-regulated cell mechanosensing is largely dependent on the responsiveness to ligand-mediated tensile/traction forces. Tensile force is generated in cell-cell or cell-matrix adhesions, which is exerted by pulling of cell surface receptors via engaged ligands. Due to the importance of tensile force-mediated cellular mechano-transduction, many approaches have been developed to measure receptor-exerted tensile forces, including traction force microscopy,^78^^,^^79^ molecular tension fluorescence microscopy (MTFM),^36^^,^^80^ and DNA-based mechanical nano-sensors.^81^, ^82^, ^83^, ^84^ The mechano-sensing mechanisms of these methods provide insights into the future development of the mechanotransduction strategy of artificial mechanoreceptors. For example, traction force microscopy (TFM) evaluates polymer substrate deformations to map cellular traction stresses, which is an indirect measurement of receptor forces and suffers from its limited spatial (micrometer) and force resolution (nN levels). Several single molecule force spectroscopy (SMFS) tools, such as atomic force microscopy (AFM),^85^, ^86^, ^87^ optical/magnetic tweezers (OT/MT),^88^^,^^89^ and the biomembrane force probe (BFP),^90^^,^^91^ exhibited improved force resolution, allowing the detection of pN-level forces exerted by individual cell surface molecules, but they are not available for high-throughput analysis. Genetically encoded protein tension sensors are compatible with high-throughput bioimaging.^92^, ^93^, ^94^ They typically function within the range of 2–6 ​pN, which is relatively small compared to the general realm of biological forces. Molecular tension fluorescence microscopy (MTFM) detects forces ranging from 1 to 20 ​pN using the fluorescence signal emitted by PEG-based probes.^80^ However, these peptide-based probes present the complexity of a graded “analog” response, complicating the interpretation of the absolute force magnitude from the fluorescence signal. In contrast, taking advantage of the flexibility and programmability of the DNA molecular switch, DNA-based mechanical sensors (such as DNA duplex and hairpin) ensure a “digital” response to a threshold discrete force value, which is highly tunable by modulating DNA structures.

#### 4.1.1 DNA duplex-based probes

**Fig. 4:**
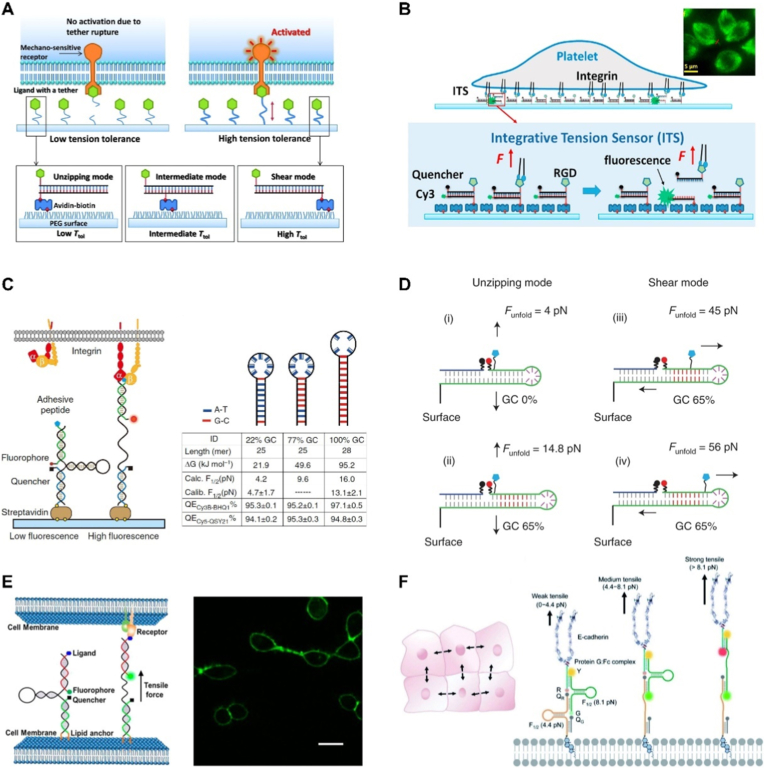
DNA duplex and hairpin-based mechanical probes for cellular tensile/traction force sensing. (A) Tension gauge tether (TGT) system based on DNA duplexes, which dissociates when the force applied by the mechanoreceptor is larger than the rupture threshold to control mechanoreceptor activation.^96^ Copyright 2013, The American Association for the Advancement of Science. (B) Application of TGT probes for mapping integrin-mediated forces in platelets by fluorescence activation that occurs upon force-induced DNA unzipping.^97^ Copyright 2017, Elsevier. (C) DNA hairpin-based force probe design with unfolding force ranging from 4 ​pN to 16 ​pN.^100^ Hairpin unfolding under tension separates fluorophore from quencher, resulting in fluorescence recovery to report integrin-mediated forces. Copyright 2014, Springer Nature. (D) Schematic of the reversible shearing DNA-based tension probe (RSDTP) with different rupture forces from 4 ​pN to 56 ​pN to track strong mechanoreceptors.^101^ Copyright 2021, Springer Nature. (E) Membrane-anchored DNA hairpin-based mechanical probes for visualizing intercellular forces.^103^ Copyright 2017, American Chemical Society. (F) DNA hairpin-based ratiometric probes for direct and precise quantification of forces at cell-cell junctions.^105^ Copyright 2010, Royal Society of Chemistry/CC BY-NC 3.0.

The DNA-based force detection technology was first proposed in 2003.^95^ In this design, the rupture force of DNA duplexes (within the pN range) served as a reference standard for measuring the mechanical strength of molecular interactions. In 2013, Wang and Ha developed the tension gauge tether (TGT) system based on DNA duplex structures (Fig. 4A).^96^ This DNA force probe features substrate-anchored ends and bio-ligand conjugated ends for cell surface receptor recognition, capable of reporting pN-scale forces exerted by cellular receptors. Cells transmit mechanical forces through membrane receptors to the ligand-coupled DNA probes, inducing DNA strand separation and conformational changes when the force is larger than the DNA rupture threshold. The DNA duplex dissociation process is digital and irreversible, inducible through either axial stretching (shear mode) or perpendicular pulling (unzipping mode). Using TGT probes with tension threshold values ranging from 12 to 58 ​pN, this study provides the first quantitative definition of the single-molecule force thresholds required for integrin and Notch activation, and establishes a broadly applicable platform for investigating mechanical signaling. By labeling DNA duplexes with a fluorophore and quencher, force-induced separation can be detected through quantifiable fluorescence changes. Monitoring these fluorescence changes via fluorescence microscopy enables high-resolution mapping of the spatial distribution of integrin-mediated forces. Wang et al. developed a TGT-based force-activated fluorescent sensor that emits fluorescence when integrin binds to RGD and applies tension exceeding a set threshold, leading to the separation of double-stranded DNA and the dissociation of fluorophore from quencher (Fig. 4B).^97^ Using this sensor, they achieved high-resolution imaging and quantification of integrin-mediated forces in platelets at the molecular level. Their work revealed the polarized distribution and biphasic force generation mechanism of platelet tension, providing a powerful tool for studying platelet mechanobiology with significant translational potential.

#### 4.1.2 DNA hairpin-based probes

Beyond DNA duplex structures, DNA hairpins represent another widely employed DNA nanostructure for constructing force-sensing probes. These hairpins exhibit rapid and reversible mechanical transitions between folded and unfolded states. Similar to DNA duplexes, DNA hairpins undergo digital structural transitions, where only forces exceeding the characteristic F_1/2_ threshold (defined as the force at which 50 ​% of hairpins unfold) can be detected.^98^ Mechanical loading induces unzipping of the stem in hairpin, forming a single-stranded DNA region that functions as an entropic spring, with spontaneous refolding occurring upon force removal, demonstrating reversible unfolding behavior distinct from the irreversible structural changes of DNA duplexes. This reversible opening-closing capability enables repeated measurements of force-induced conformational changes on individual target molecules, which proves essential for the comprehensive investigation of folding/unfolding dynamics and ensures experimental accuracy in single-molecule studies. Importantly, the force-responsive characteristics of both DNA duplexes and hairpins can be precisely calculated and predicted, with straightforward tunability achieved through modifications to nucleotide sequences or adjustments of stem/loop lengths.

In 2006, Block et al. conducted systematic mechanical and thermodynamic studies on DNA hairpins, establishing the theoretical foundation for calculating force thresholds in various DNA hairpin-based force probes.^98^ In 2007, Lang et al. achieved the first real-time monitoring of force-induced DNA hairpin unfolding at single-molecule resolution by integrating DNA hairpins with fluorescent reporters using Förster resonance energy transfer (FRET) technology.^99^ Building upon this advancement, Salaita et al. developed a more convenient and cost-effective DNA hairpin force sensor system.^100^ This innovative sensor comprises three key components, including a surface-anchored strand labeled with quencher, a fluorophore-modified ligand strand, and a complementary hairpin strand (Fig. 4C). In the resting state, fluorescence remains quenched due to close proximity between the fluorophore and quencher. When cellular receptors recognize the ligand and apply tension exceeding the F_1/2_ threshold (normally 4−16 ​pN), hairpin unfolding separates the fluorophore-quencher pair, resulting in fluorescence recovery. This study achieved high-resolution, quantitative, and dynamic imaging of integrin-transmitted pN-scale forces, revealing the force dynamics, ligand selectivity, and heterogeneity of force distribution within focal adhesions during early adhesion. Therefore, it provides a powerful tool for investigating cellular mechanotransduction.

Although DNA-based mechanical probes have achieved significant results in the analysis of biological forces, the aforementioned probes are incapable of reversibly measuring mechanical forces at higher force levels (greater than 20 ​pN). To overcome this limitation, Liu et al. developed a reversible shearing DNA-based tension probe (RSDTP) for detecting molecular pN-scale forces ranging from 4 to 60 ​pN transmitted by cells, revealing the critical role of high-strength integrins in focal adhesion maturation and mechanotransduction (Fig. 4D).^101^ Building on this, they further created the ForceChrono probe, which captures the temporal dynamics of mechanical forces (duration, loading rate, etc.).^102^ This probe employs a dual-hairpin structure with distinct force thresholds, calculating the loading rate based on the time interval between the sequential unfolding of the two hairpins and measuring force duration via the duration of luminescence. For the first time, simultaneous measurement of single-molecule force magnitude, duration, and loading rate has been achieved in living cells, uncovering a positive feedback mechanism between the mechanical stability of integrin-ligand bonds and force magnitude. This advancement holds broad application potential for future research on mechanical signal decoding in processes such as immune recognition, stem cell differentiation, and tumor metastasis.

While the cellular mechanics studies primarily focus on cell-extracellular matrix interactions, mechanical signal transduction through intercellular junctions plays an equally crucial role in cell communication within native multicellular environments. You et al. recently developed a membrane-anchored DNA hairpin tension probe (MDTP) consisting of three functional components: a hairpin strand serving as the mechanical sensing module, a ligand strand for recognizing adjacent cell receptors, and a lipid-modified strand for cell membrane anchoring (Fig. 4E).^103^^,^^104^ When mechanical binding occurs between intercellular ligand-receptor pairs, the DNA hairpin structure unfolds to activate fluorescent signals, which enables direct visualization of intercellular tension mediated by integrin and E-cadherin, and reveals mechanical differences across various types of cell junctions. To further achieve quantitative measurement of intercellular tension, their team subsequently developed a second-generation probe termed DNA Membrane Tension Ratio Probe (DNAMeter) (Fig. 4F).^105^ This advanced probe incorporates two self-assembled hairpin structures and two orthogonal fluorophore-quencher pairs, enabling simultaneous detection of mechanical signals at different magnitude ranges. By introducing reference dyes for normalizing probe membrane distribution and analyzing intensity ratios between reference and reporter fluorescence, this system achieves quantitative assessment of intercellular tension across a broad spectrum. Using E-cadherin-mediated epithelial cell junctions as a model, the technology precisely quantifies tension distribution across three distinct ranges: <4.4 ​pN, 4.4–8.1 ​pN, and >8.1 ​pN, demonstrating remarkable precision in mapping mechanical microdomains at cell-cell interfaces. These probes hold future potential for application in the study of collective cellular processes, such as cancer cell invasion, wound healing, and embryonic development.

Over the past few decades, multiple cellular force detection technologies have matured successively. Among these, DNA-based force probes have emerged as essential biophysical tools for studying cellular mechanotransduction due to their unique advantages. Continued innovation in this field promises to deepen our understanding of molecular biophysics in developmental biology, immunology, cancer biology, and related disciplines.

### 4.2 DNA-based mechanical regulation

**Fig. 5:**
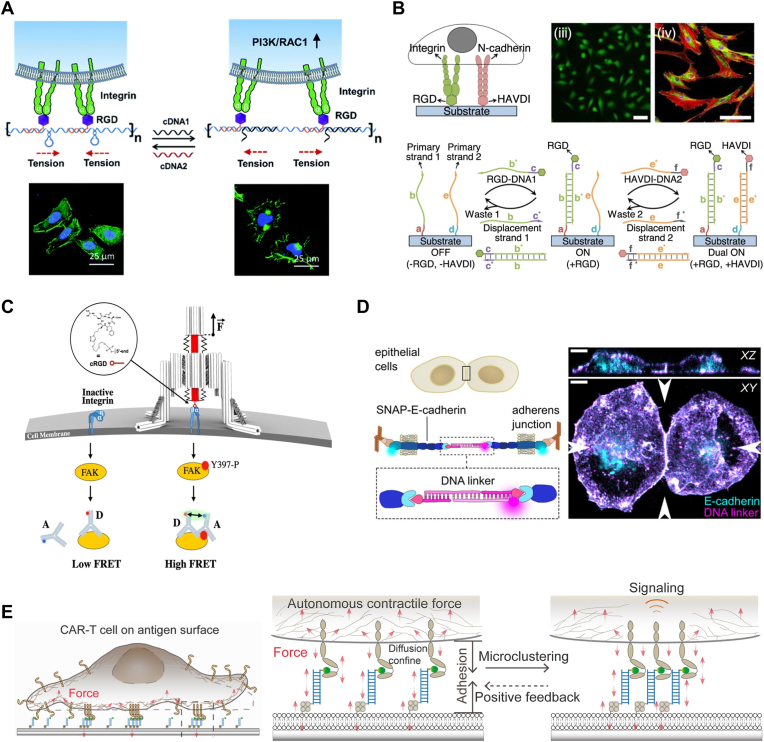
DNA mechanosensitive nanodevices for modulation of mechanoreceptors. (A) DNA hairpin-based spring for dynamic regulation of integrin clustering via strand displacement reactions.^106^ Copyright 2010, Royal Society of Chemistry/CC BY 3.0. (B) Force ligand positioning platform for nanoscale control of reversible cell adhesion and spreading via orthogonally presenting RGD and HAVDI peptides.^109^ Copyright 2022, Springer Nature/CC BY 4.0. (C) Nano-winch, a DNA origami-based actuator, applies pN-scale forces to integrins to induce downstream signaling, including focal adhesion kinase phosphorylation.^110^ Copyright 2022, Springer Nature/CC BY 4.0. (D) The DNA-E-cadherin hybrid system uses complementary DNA duplexes to regulate intercellular adhesion strength and reversibility via strand displacement.^111^ Copyright 2021, American Chemical Society/CC BY 4.0. (E) DNA duplex-based mechanical ligand presentation system for quantifying the role of force magnitude in immune receptor micro clustering and signal activation.^113^ Copyright 2024, Elsevier.

Mechanical forces play pivotal regulatory roles in cellular communication with both the microenvironment and neighboring cells. Critical biological processes, including cell proliferation, morphological changes, migratory behaviors, tissue homeostasis, and morphogenesis, are precisely governed by mechanical signaling. Upon environmental stimulation, cell membrane receptors detect ligand-mediated mechanical forces to modulate signal transduction and behavioral responses. DNA-based mechanosensitive nanodevices for mechanoreceptor manipulation have emerged as indispensable tools for both decoding and manipulating mechanotransduction pathways in contemporary cell mechanics research (Fig. 5).

#### 4.2.1 Force signaling regulation of integrin

Leveraging the predictable Watson-Crick base pairing and structural programmability, DNA-based dynamic reactions and self-assembled nanostructures demonstrate exceptional potential for flexible regulation of mechanoreceptors. Integrins, serving as pivotal mechanosensors mediating cellular adhesion and force response to ECM, can be precisely controlled at the nanoscale through spatial organization of their functional RGD ligands. Li et al. developed a DNA hairpin-based nano-spring with multivalent RGD ligands that enable dynamic and reversible regulation of integrin clustering states on cell membranes (Fig. 5A).^106^ This system utilizes rolling circle amplification (RCA) to synthesize multivalent ligand scaffolds and precisely controls nanoscale receptor arrangement through strand displacement reactions, thereby regulating integrin signaling pathways and cell morphology. The results demonstrate that the motion of DNA nano-springs can significantly influence the development of cellular protrusions and alter mRNA expression levels associated with integrin clustering. Mounting evidence suggests that the activation of mechanosensitive receptors is highly dependent on their nanoscale organization. Therefore, controlling the spatial distribution of cell surface receptors is crucial for both studying fundamental signaling pathways and externally manipulating cellular behavior. Furthermore, given the excellent versatility of DNA nanotechnology, additional functional modules responsive to pH or light could be incorporated to achieve spatiotemporal control of integrin receptor organization.^107^^,^^108^ These approaches have the potential to provide a dynamic and controllable system for investigating integrin-related mechanics in living cells.

DNA molecular tools can also regulate integrin-mediated cell adhesion behaviors by adjusting the orthogonal and reversible presentation of mechanical functional ligands in the culture environment. Lin et al. described a molecular positioning system with nanometer precision to control reversible cellular functions (Fig. 5B).^109^ This platform can programmatically present RGD peptides that specifically bind integrins and HAVDI peptides that bind N-cadherin, respectively mimicking cell-extracellular matrix interactions and cell-cell interactions, while utilizing DNA hybridization and strand displacement reactions to regulate cell adhesion and reversible spreading over multiple cycles. For the first time, this platform achieved the simulation of dynamic transition processes between cell-ECM interactions and cell-cell interactions in vitro, providing a universal and programmable tool and mechanistic framework for studying the regulation of stem cell fate by dynamic mechanical microenvironments in development, injury repair, and tissue engineering.

In addition to regulating the spatial organization of receptors and ligands, DNA nanotechnology can also be directly employed to modulate the activation of integrin signaling pathways. For instance, Bellot et al. introduced a robust, easily assembled, and programmable DNA origami-based molecular actuator termed the Nano-winch (Fig. 5C).^110^ The Nano-winch applies finely tuned pN-scale mechanical forces in autonomous and remote activation modes to manipulate multiple mechanoreceptors in parallel via adjustable single- and double-stranded DNA linkages. The Nano-winch can land on cell surfaces and target integrins, exerting mechanical forces to induce conformational changes, thereby stimulating detectable downstream focal adhesion kinase phosphorylation. This demonstrates that a DNA-based nanodevice can be applied to regulate cellular mechanotransduction processes.

#### 4.2.2 Force signaling regulation of cadherin

E-cadherin-mediated intercellular adhesion is crucial for regulating tissue integrity, signal transduction, and collective cell dynamics. Cavalcanti-Adam et al. pioneered a DNA-E-cadherin hybrid system as a tool for precisely modulating the adhesive strength between epithelial cells (Fig. 5D).^111^ In this system, complementary DNA strands are covalently conjugated to the extracellular domains of E-cadherin to regulate cell-cell binding behavior. The programmable sequence design of DNA allows flexible adjustment of the binding strength of DNA-E-cadherin hybrid molecules, while rapid and reversible regulation can be achieved through strand displacement reactions. The DNA-E-cadherin hybrid system promotes strong and reversible cell-cell adhesion in E-cadherin-deficient cells by forming adherent junctions. This strategy enables direct assessment of how cell-cell adhesion strength influences intracellular signaling and collective cell dynamics while preserving downstream signaling integrity. It provides a versatile tool for studying adhesion-dependent tissue homeostasis, signaling pathways, and cancer invasion, demonstrating the potential of DNA nanotechnology in engineering mechanical interactions within cell populations.

#### 4.2.3 Force signaling regulation of TCR

In adaptive immunity, mechanical forces facilitate T cell activation by participating in the TCR signaling transduction process. Douglas et al. developed a DNA origami-based system that enables flexible modulation of TCR spatial organization and regulation of cellular signaling.^112^ This system replaces conventional pMHC-TCR interactions with DNA duplex hybridization, allowing nanoscale control over ligand patterns and binding affinities on DNA origami to investigate how TCR spatial arrangement influences signaling kinetics. This work demonstrates the versatility of DNA nanotechnological tools in dissecting and manipulating TCR-mediated mechanotransduction mechanisms. More recently, Xu et al. employed a DNA duplex-engineered mechanical ligand presentation platform to elucidate how mechanical force magnitude influences receptor micro clustering, signal transduction, and immune responses (Fig. 5E).^113^ This work quantitatively delineated the pivotal regulatory role of mechanical forces in immune receptor synapse formation, providing a programmable mechanical framework for mechanoimmunology research.

### 4.3 De novo DNA-functionalized artificial mechanoreceptors for mechanotransduction regulation

The generalized engineering strategy for constructing synthetic mechanoreceptors largely relies on sophisticated genetic engineering of natural mechanoreceptors. Considering the limitation in the type of natural mechano-switch scaffolds, it is crucial to develop an adaptable, chemobiological approach available for receptor mechanoresponsive modification independent of natural mechanosensory mechanisms. DNA-based mechanosensitive nanodevices have made significant progress in manipulating natural mechanoreceptors, presenting a valuable toolkit for rewiring natural mechanical signaling pathways in living systems. However, to date, there has been no research attempt to employ DNA mechanosensitive nanodevices to endow non-mechanosensitive receptors with force-responsiveness.

**Fig. 6:**
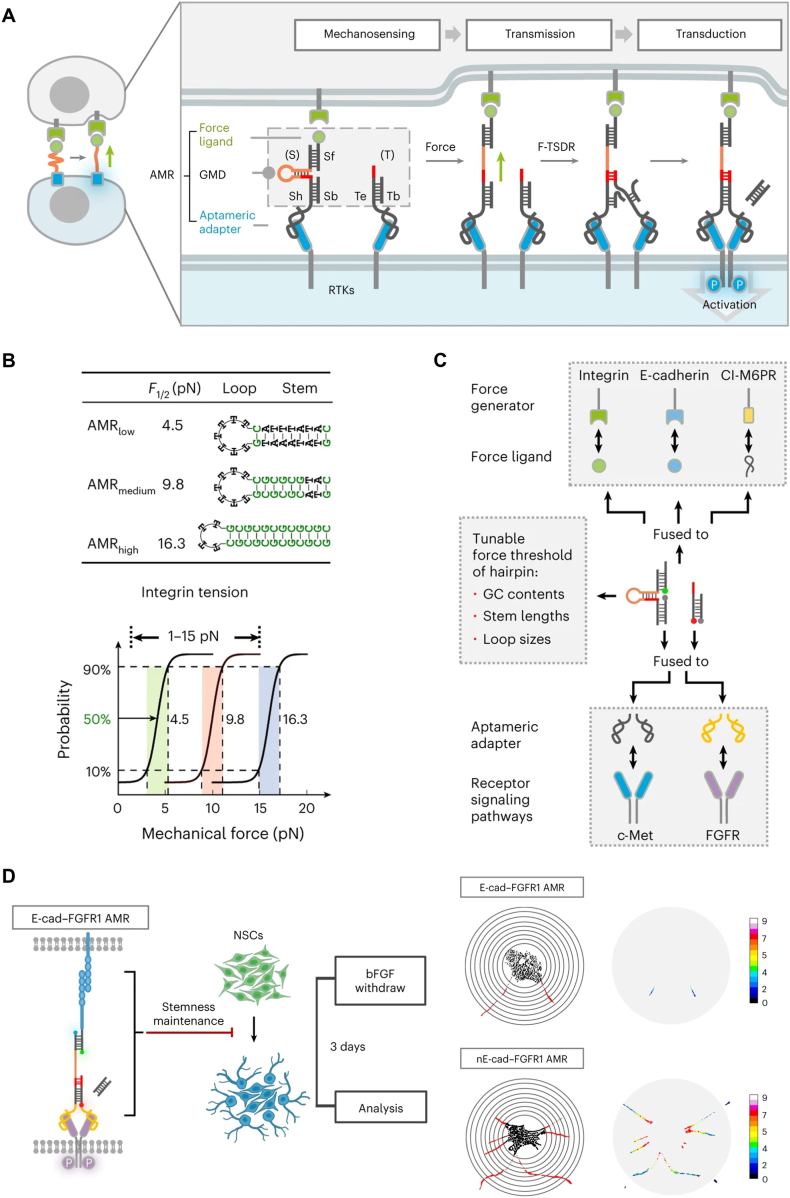
De novo AMRs for cellular mechanotransduction regulation.^32^ (A) Schematic of AMR design. A GMD nanodevice was nongenetically functionalized on native non-mechanosensitive RTKs via aptamer anchors to constitute the AMRs, which sense force via DNA hairpin-based allosteric switch, thereby implementing DNA strand displacement to promote RTK dimerization and downstream signaling. (B) Programmable DNA hairpin sequences enable tunable mechanical response thresholds with pN-scale resolution. (C) Versatility of AMRs to respond to diverse mechanical inputs and reprogram different receptor signaling pathways. (D) Rewiring FGFR1 signaling into a force-responsive circuit to maintain neural stem cell stemness via mechanical stimulation. Copyright 2024, Springer Nature.

In this context, our group developed a de novo DNA-functionalized artificial mechanoreceptor (AMR) that offers a novel nongenetic alternative method to endow native receptors with force-responsiveness in a plug-and-play manner via chemical functionalization (Fig. 6A).^32^ The AMR represents a highly modular DNA-protein chimera featuring a generalized mechanosensing-and-transmitting DNA (GMD) nanodevice, which enables force-induced signal transduction by functionalizing native cell surface RTKs via aptameric anchors. Our DNA-based AMR takes advantage of a tunable and predictable thermodynamic property of DNA hybridization and the high programmability and functionalization of DNA dynamic reactions, which allows for rational design-based receptor construction that precisely controls mechanoresponsive properties and threshold ranges. Therefore, by simply programming the DNA hairpin sequences, the force threshold of DNA mechanical switches can be finely tuned with pN resolution (Fig. 6B). Thus, this approach enables precise calibration of AMR force sensitivity to filter incoming cellular forces and achieve digitalized signal responses. Furthermore, by coupling diverse force-sensing ligands, including peptides, antibodies, and DNA aptamers, the AMR can respond to multiple cellular force inputs. These mechanical inputs encompass but are not limited to actin-generated pulling forces mediated by adhesion receptors (integrins, E-cadherin) and clathrin-mediated endocytic forces generated during membrane protein (Cl-M6PR) internalization (Fig. 6C).^114^^,^^115^ Remarkably, the AMR can chemically functionalize various force-insensitive receptors as mechanoresponsive signal outputs through simple exchange of specific DNA aptamer domains, demonstrating its versatile programmability. We successfully rewired the FGFR1 pathway into a force-responsive circuit,^116^ achieving mechanical force-driven maintenance of neural stem cell stemness (Fig. 6D).^117^^,^^118^ This pioneering work establishes, for the first time, a new paradigm for constructing artificial mechanical receptors independent of natural mechanosensing mechanisms. Together, in comparison with conventional genetic engineering strategies, the non-genetic engineering approach of AMR represents a promising alternative strategy with unique features in the field of synthetic mechanoreceptor engineering.

This AMR strategy can repurpose non-mechanosensitive receptors to sense new mechanical signals, opening several promising future directions. One potential future direction involves integrating force signals with other input signals, such as ligands,^119^^,^^120^ light,^121^ or ultrasound,^122^^,^^123^ to advance the development of multi-input, logic-gated signaling networks,^124^ thereby enabling high-precision control over cellular signaling pathways. Moreover, given the excellent applicability of the AMR approach, future efforts may focus on designing DNA nanodevices to reprogram oligomerization-regulated signaling in other types of receptors, such as G protein-coupled receptors^125^ and tumor necrosis factor receptors.^126^ Additionally, the chemically synthetic and non-genetic nature of AMR allows it to be readily integrated with existing synthetic receptor systems, including dimerization-activated platforms such as MESA,^127^ GEMS,^128^ and dCas9-synRs,^129^ for constructing semi-synthetic AMRs with orthogonal sensing and response capabilities, leading to more efficient, specific, and customizable regulation of endogenous signaling pathways. Thus, DNA-based mechanosensitive nanodevices and artificial mechanoreceptor reprogramming technologies will lay an important foundation for developing novel chemical biology tools to precisely manipulate a broader range of mechanotransduction pathways in the future.

## 5 Conclusion

This review summarizes recent advances in mechanoreceptor engineering from conventional genetic receptor engineering to DNA nanotechnology-based non-genetic approaches. We first elucidate the operational principles of natural mechanoreceptors, followed by a discussion of achievements in genetic-encoding strategies for receptor modification and reprogramming. Genetically engineered synthetic mechanoreceptors, including synNotch and synCAMs, have demonstrated exciting successes in tissue patterning and immunotherapy by customizing force-input to signal-output programs through genetic modifications of their native mechanical architectures. However, their reliance on natural mechanosensing mechanisms limits their generalizability to non-mechanosensitive receptors. Moreover, genetic strategies face laborious optimization processes and potential risks associated with exogenous gene expression.

In contrast to conventional genetic-encoding strategies, DNA-based non-genetic engineering represents a paradigm shift, offering highly programmable, modular, and precise tools for manipulating cellular mechanotransduction. DNA mechanosensitive nanodevices, such as DNA duplex-based tether, DNA hairpin-based spring, DNA origami-based scaffold and actuator, enable real-time, high-resolution control over receptor organization, force sensing, and signaling activation without genetic alteration. A flagship innovation is the AMR, which functionalizes non-mechanosensitive receptors (e.g., RTKs) via aptamer anchoring and DNA allosteric switches, enabling de novo force-responsive signaling and customized cellular behaviors such as mechanical maintenance of stemness.

Looking ahead, DNA-based receptor non-genetic engineering faces challenges in customizing intracellular signal rewiring and achieving orthogonality with endogenous pathways. Future integration of DNA nanodevices with synthetic receptor systems may generate semi-synthetic mechanoreceptors to achieve ideal orthogonal sensing and mechanotransduction capabilities, enabling high-precision cellular programming. Additionally, integrating mechanical signals with multimodal inputs such as light, sound, and chemical ligands to construct logic-gated signaling networks will also be an important direction for future research.

In summary, DNA nanotechnology has profoundly expanded the toolbox for mechanobiology, providing a non-genetic, tunable, and versatile platform to reprogram cellular mechanotransduction. As these technologies continue to evolve, they hold exceptional promises for pioneering force-directed cell therapies and opening new avenues in regenerative medicine, immunotherapy, and fundamental biological research.

## CRediT authorship contribution statement

**Sihui Yang:** Writing – original draft, Validation, Supervision, Resources, Investigation, Funding acquisition, Formal analysis, Conceptualization. **Zhou Nie:** Writing – review & editing, Validation, Supervision, Resources, Investigation, Funding acquisition, Formal analysis, Conceptualization.

## Ethical approval

This study does not contain any studies with human or animal subjects performed by any of the authors.

## Declaration of competing interest

The authors declare that they have no known competing financial interests or personal relationships that could have appeared to influence the work reported in this paper.
